# Elucidating the nature of acinic cell carcinoma of the breast with high-grade morphology: evidence from case report

**DOI:** 10.1186/s13000-024-01521-1

**Published:** 2024-07-24

**Authors:** Yunjie Ge, Xianping Wei, Jing-Nan Liu, Ping-Li Sun, Hongwen Gao

**Affiliations:** 1 Department of Pathology, The Second Hospital of Jilin University, Changchun, 130022 China; 2 Department of Clinical Research, The Second Hospital of Jilin University, Changchun, China; 3 Department of Respiratory Medicine, The First Affiliated Hospital of Jilin University, Changchun, China

**Keywords:** Acinic cell carcinoma, Triple-negative breast carcinoma, High-grade morphology, Next‑generation sequencing, High-grade transformation

## Abstract

**Background:**

Acinic cell carcinoma (AciCC) of the breast is a rare subtype of breast cancer. It was considered a low-grade triple-negative breast cancer (TNBC) with the potential to progress or transform into a high-grade lesion because of the molecular similarities with conventional aggressive TNBC in several genetic studies. Microscopically, the coexistence of classical low-grade and high-grade triple-negative components in breast AciCC is not uncommon. However, there is a scarcity of research on the comparative histopathological and genetic aspects of both components.

**Case presentation:**

A 34-year-old woman with a nontender mass in the upper outer quadrant of the left breast was initially diagnosed with a malignant small round cell tumor (undifferentiated or poorly differentiated carcinoma) based on a preoperative biopsy, which was later identified as breast AciCC with a high-grade solid component. Left breast-conserving surgery with sentinel lymph node biopsy was performed. Microscopically, the breast AciCC consisted of a classical acinic component and a high-grade component. The latter demonstrated a solid sheet-like pattern characterized by large, round, pleomorphic or vesicular nuclei, prominent nucleoli, and frequent mitotic activities. Classical acinic architectures focally merged together to form solid nests and transited into high-grade areas. Remarkably, in the high-grade lesion, conventional immunochemical markers for breast AciCC, such as α1-antitrypsin (AAT), Lysozyme (LYS), Epithelial membrane antigen (EMA), S100 protein (S100), and cytokeratin (CK) were negative, whereas cell cycle protein D1 (cyclin D1) and vimentin showed diffuse expression. Next‑generation sequencing (NGS) revealed that 43.5% of variants were identical in both components. Furthermore, *PAK5* mutation; copy number (CN) loss of *CDH1*, *CHEK1*, and *MLH1*; and CN gains of *CDK6*, *HGF*, and *FOXP1* were identified in the high-grade lesion. The patient was treated with eight cycles of adjuvant chemotherapy (epirubicin combined with cyclophosphamide) and radiotherapy after surgery, and she is currently alive for 43 months with no metastases or recurrences.

**Conclusions:**

This case demonstrates a comparative analysis of the histopathological and genetic characteristics of classical low-grade and high-grade components of AciCC within the same breast. This information may serve as a morphological and molecular basis for further investigation into the molecular mechanisms underlying high-grade lesions in breast AciCC.

## Background

Acinic cell carcinoma (AciCC) of the breast is a rare malignant epithelial tumor that was first reported in 1996 [1] and is classified as a rare and salivary gland-type tumor by the 5th World Health Organization classification of tumors of the breast [2]. Although the histological patterns of breast AciCC overlap with those of salivary AciCC, studies have demonstrated that they have different molecular underpinnings [3, 4]. Some researchers have proposed that breast AciCC should be considered a type of carcinoma developing in microglandular adenosis (MGA) with acinic cell differentiation, rather than as a distinct entity [5]. Moreover, there is evidence that the morphological and immunohistochemical transition from typical MGA to atypical MGA and ultimately to AciCC or poorly differentiated components can occur [6, 7]. Subsequent genetic studies have supported the contention that MGA, atypical MGA, and AciCC may be a part of the same spectrum of lesions and may represent low-grade forms of triple-negative breast cancer (TNBC) with the potential to progress or transform into high-grade TNBC [8].

Additionally, breast AciCC was previously suggested to have a good prognosis. Nonetheless, several cases with recurrence, metastases, or death have been reported successively [9, 10]. Hence, the true nature and origin of breast AciCC remain yet to be clarified, especially cases with high-grade morphology. Herein, we conducted a comparative investigation of the histopathological and molecular features of low-grade and high-grade components in a single case of breast AciCC. Our aim was to provide a morphological and molecular basis for further exploration of the molecular mechanisms underlying high-grade lesions in breast AciCC.

## Case presentation

**Fig. 1:**
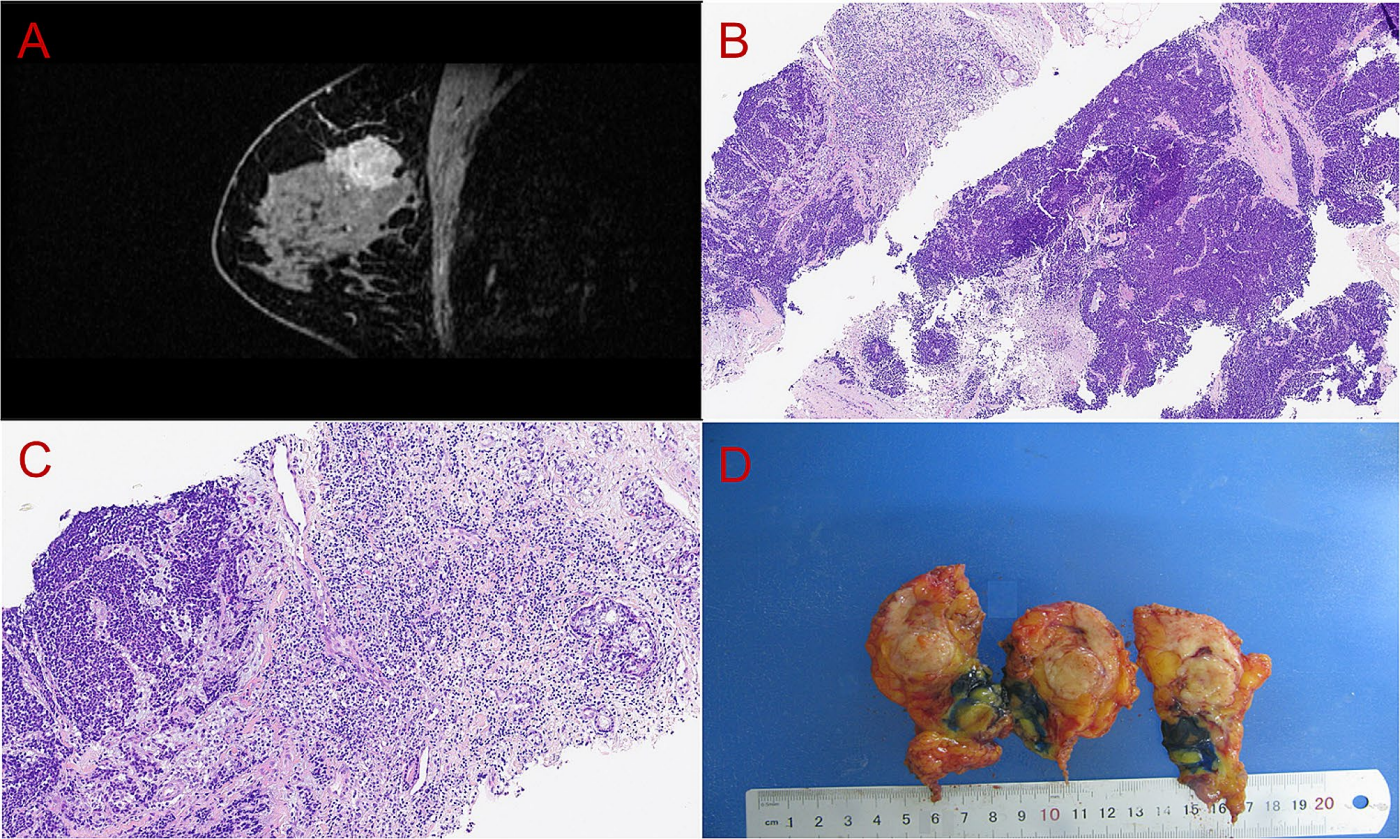
Imageological and pathological examination. Enhanced magnetic resonance imaging revealed an irregular mass in the left breast (**A**). Diffuse solid sheet-like tumor nests, focal acinic component, and abundant lymphocytes were observed in preoperative core needle biopsy (**B**, H&E 40×; **C** H&E, 100×). Gross examination revealed a solid nodule (**D**). (H&E, hematoxylin and eosin.)

A 34-year-old woman discovered a painless mass through self-palpation in the left breast for 5 days. Physical examination revealed a hard mass in the upper outer quadrant of the left breast, measuring 30 mm in diameter. She was previously healthy and denied a family history of cancer. The nipple and the skin overlying the mass were normal. There were no palpable axillary and supraclavicular lymph nodes. Ultrasonography revealed an irregular hypoechoic mass in the upper outer quadrant of the left breast (BIRADS: 4B), and enhanced magnetic resonance imaging revealed an ill-defined, roundish signal shadow (BIRADS: 6) (Fig. 1A). Based on preoperative core needle biopsy, the diagnosis was a malignant small round cell tumor (undifferentiated or poorly differentiated carcinoma) (Fig. 1B, C). We performed modified radical mastectomy and axillary lymph node excision on the patient. Intraoperative sentinel lymph nodes and surgical margins were negative.

### Clinicopathological examination

**Fig. 2:**
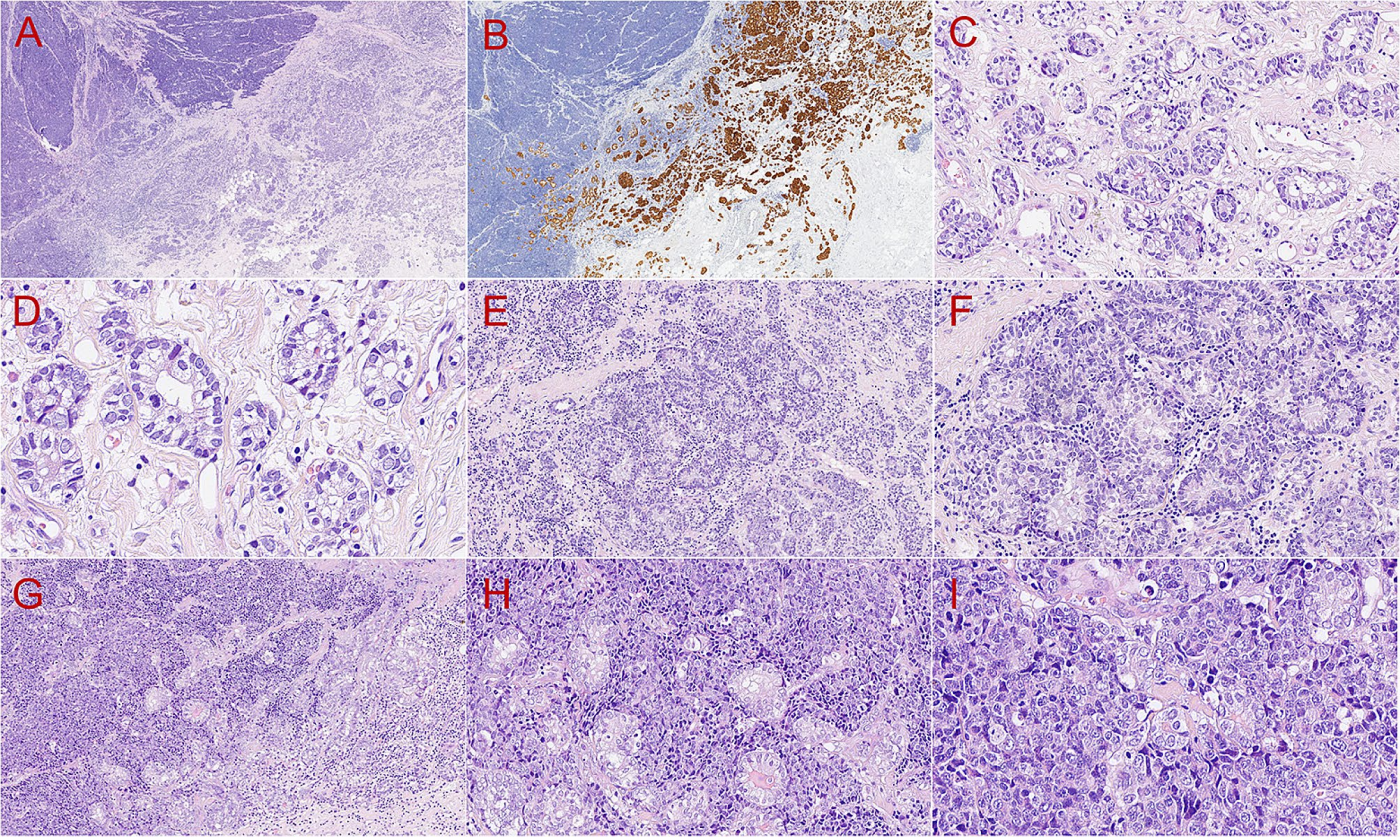
Histopathological features of the tumor. Microscopically, the tumor consisted of a classical acinic component and a solid high-grade component (**A**, H&E, 20×). SOX10 (**B**, EnVision, 20×) staining highlighted the distribution of the two components. The acinar architectures arranged irregularly were composed of one to several layers of neoplastic epithelial cells (**C**, H&E, 200×). There were no myoepithelial cells or basal lamina, and neoplastic glands were focally surrounded by a capillary network (**D**, H&E, 400×). Between both components, acinic architectures merged together into small solid or cribriform nests, and the number of stromal tumor-infiltrating lymphocytes was high (**E**, H&E, 100×; **F**, H&E, 200×). The transition from neoplastic glands or confluent nests to the large solid region (**G**, H&E, 100×; **H**, H&E, 200×; **I**, H&E, 400×) and the faint outline of the remaining glands in the high-grade lesion (I, H&E, 400×) were observed

Grossly, the tumor was a grayish-white, well-defined nodule with soft texture and measured 27 mm in its greatest axis (Fig. 1D). Microscopically, the tumor grew infiltratively and comprised a classical breast AciCC component and a solid sheet-like architecture with necrosis (Fig. 2A). SRY-box transcription factor 10 (SOX10) staining revealed the distribution of two components (Fig. 2B). The classical AciCC component was composed of irregular acinar or glandular architectures lined by one to several layers of neoplastic epithelial cells (Fig. 2C). Neoplastic cells in the classical AciCC component were round or cuboidal in shape with eosinophilic granular cytoplasm and unclear cytoplasmic borders (Fig. 2D). There were no myoepithelial cells or basal lamina, and neoplastic glands were focally surrounded by a capillary network (Fig. 2D). Nuclei were round, oval-to-irregular with granular chromatin. Eosinophilic secretions were observed in the lumen (Fig. 2D), and approximately three mitotic counts were detected per 10 high-power fields. Between the two components, some acinic architectures merged together into small solid or cribriform nests (Fig. 2E, F). We also detected the transition from neoplastic glands or confluent nests to the large solid region (Fig. 2G, H, and I) and the faint outline of the remaining glands in the high-grade lesion (Fig. 2I). Furthermore, a large number of stromal tumor-infiltrating lymphocytes were observed in acinic and transition areas (Fig. 2E, F, and G).

**Fig. 3:**
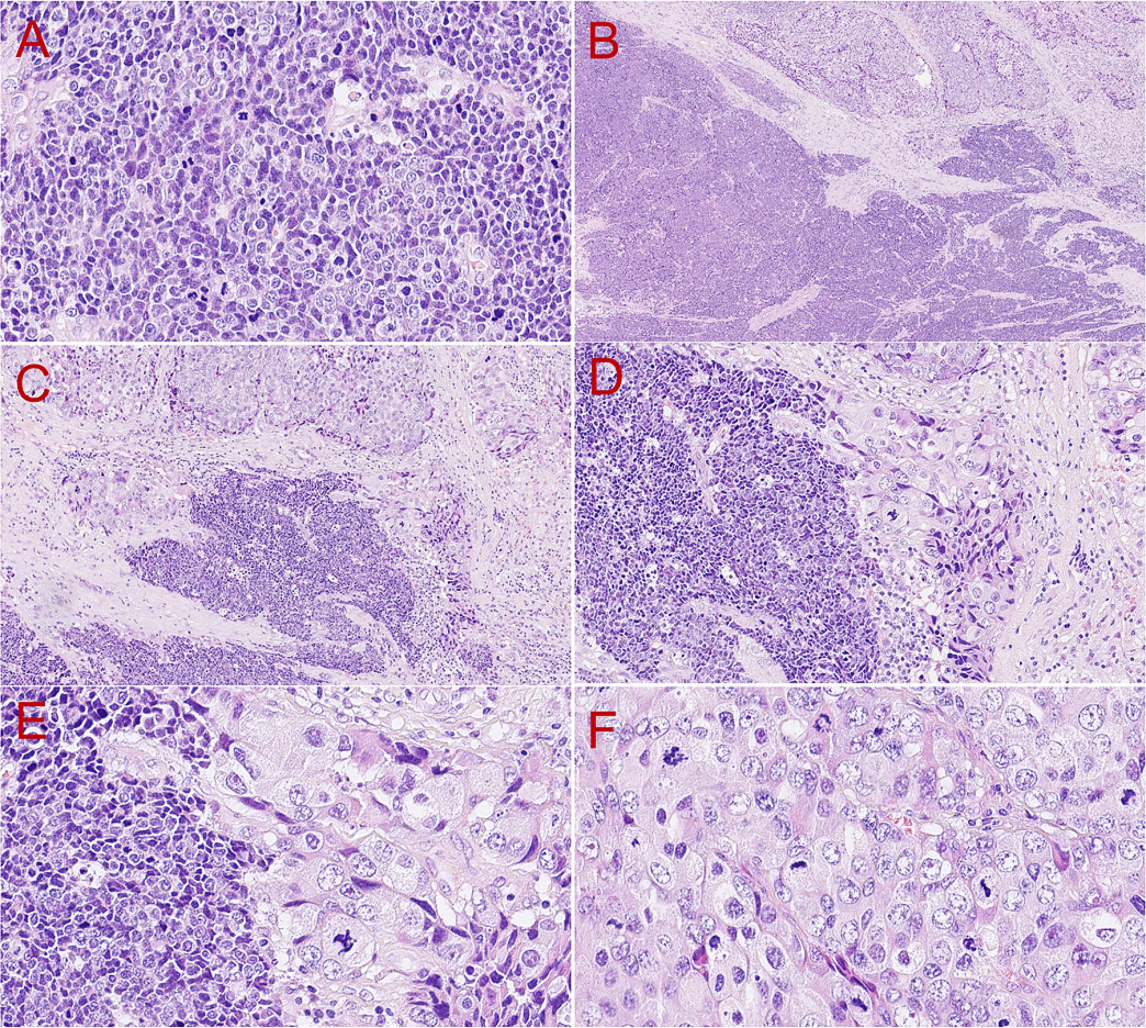
Histopathological features of the high-grade component of the tumor. The high-grade lesion exhibited a solid sheet-like pattern with marked cell atypia, weak eosinophilic cytoplasm, and round, ovoid, or polygonal nuclei (**A**, H&E, 400×). In the focal high-grade region, the tumor gradually transited into the lesion with larger vesicular pleomorphic nuclei, more prominent red nucleoli, frequent mitotic activities, and abundant cytoplasm (**B**, H&E, 40×; **C**, H&E, 100×; **D**, H&E, 200×; **E**, H&E, 400×; **F**, H&E, 400×)

In the high-grade region, the tumor exhibited a diffuse solid sheet-like pattern with marked atypia (Fig. 3A). Neoplastic cells had weak eosinophilic cytoplasm and round, ovoid, or polygonal nuclei (Fig. 3A). These round or ovoid nuclei contained coarse granular chromatin and a distinct perinuclear halo, and pleomorphic nuclei were deeply stained with little cytoplasm (Fig. 3A). In the high-grade region, there were up to 20 mitotic counts per single high-power field, and the Ki-67 labeling index was 95%. Interestingly, neoplastic cells in the focal high-grade region gradually transited into the lesion with larger vesicular pleomorphic nuclei, more prominent red nucleoli, frequent mitotic activities, and abundant cytoplasm (Fig. 3B, C, D, E, and F).

**Fig. 4:**
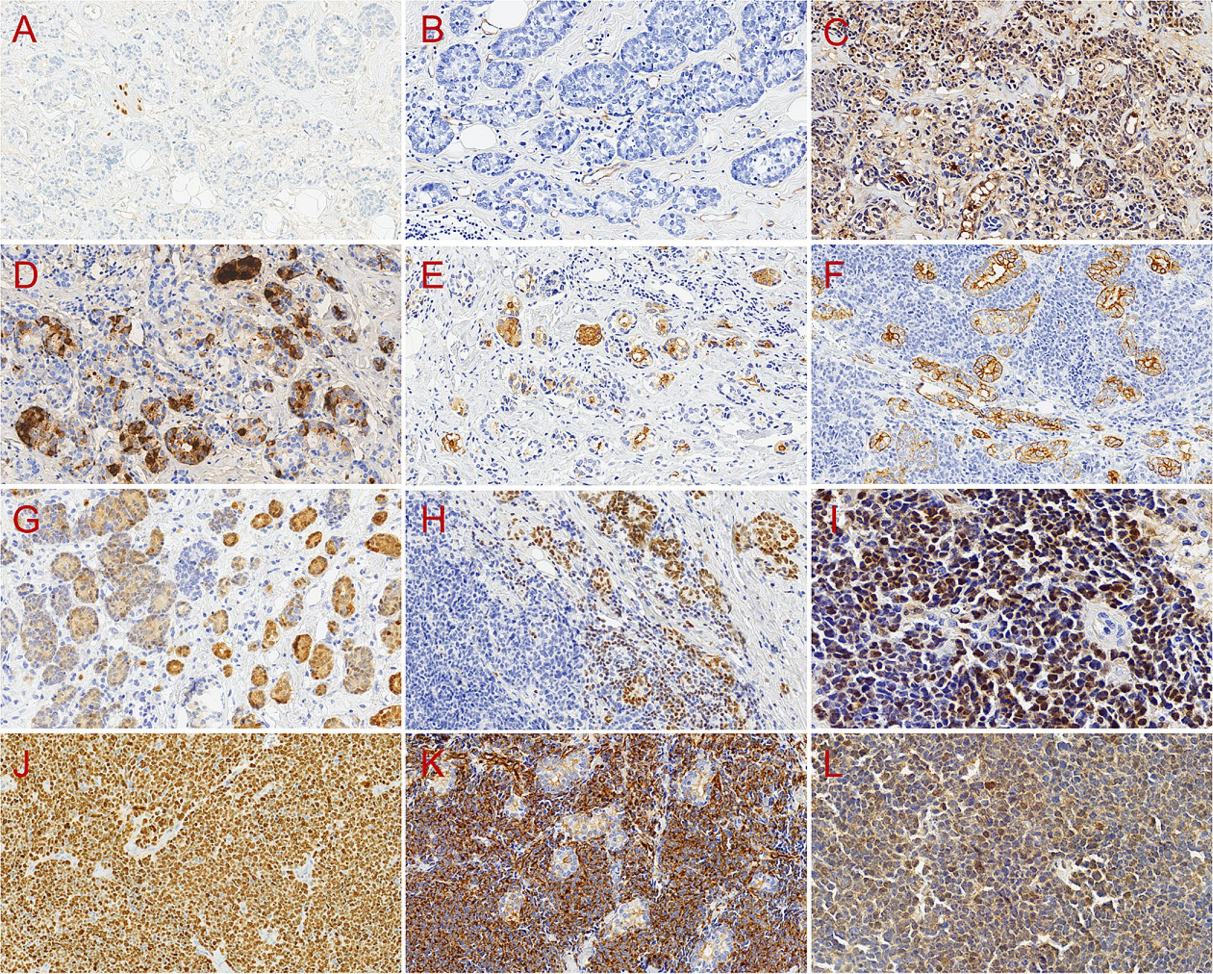
Immunohistochemical features of the tumor. The acinic component was negative for ER (**A**, EnVision, 200×). Collagen type IV (**B**, EnVision, 200×) staining revealed the absence of basement membrane around the neoplastic glands and solid nests and the presence of capillaries. And α1-antitrypsin (AAT) (**C**, EnVision, 200×), lysozyme (LYS) (**D**, EnVision, 200×), Epithelial membrane antigen (EMA) (**E**, EnVision, 200×), CK (AE1/AE3) (**F**, EnVision, 200×), S100 (**G**, EnVision, 200×), and GATA3 (**H**, EnVision, 200×) were expressed merely in the acinic component, but their expression was absent in the high-grade lesion. Cyclin D1 (**I**, EnVision, 400×) showed diffuse expression, and the Ki-67 labeling index (**J**, EnVision, 200×) was 95% in the high-grade lesion. Vimentin (**K**, EnVision, 200×) showed diffusely positive expression, and p53 (**L**, EnVision, 400×) staining was approximately 80% positive in the high-grade area

Immunohistochemically, neoplastic cells in both solid and acinic components showed a lack of expression of oestrogen receptor (ER) (Fig. 4A), progesterone receptor (PR), and human epidermal growth factor receptor 2 (HER2) and were negative for smooth muscle actin (SMA), cytokeratin 5/6 (CK5/6), p63, calponin, Gross cystic disease fluid protein 15 (GCDFP-15), Delay Of Germination 1 (DOG1), Insulinoma-associated protein 1 (INSM1), chromogranin A (CgA), Synaptophysin (Syn), CD56, and leukocyte common antigen (LCA) without mismatch repair (MMR) protein deficiency (Table 1). Collagen type IV (Fig. 4B) staining revealed the absence of basement membrane around the neoplastic glands and solid nests and the presence of abundant capillaries. Remarkably, α1-antitrypsin (AAT) (Fig. 4C), lysozyme (LYS) (Fig. 4D), Epithelial membrane antigen (EMA) (Fig. 4E), cytokeratin (CK) (AE1/AE3) (Fig. 4F), SOX10 (Fig. 1B), S100 protein (S100) (Fig. 4G), and periodic acid–Schiff-diastase (PASD) showed expression only in the classical acinic component. GATA-binding protein 3 (GATA3) (Fig. 4H) expression was detected in the classical acinic component and in scattered neoplastic cells of the solid component. Cell cycle protein D1 (cyclin D1) (Fig. 4I) showed diffuse expression, and the Ki-67 labeling index was 95% in the high-grade lesion (Fig. 4J). E-cadherin membrane expression was lost, vimentin expression was diffusely positive (Fig. 4K), and the positive rate of p53 staining was approximately 80% (Fig. 4L) in the high-grade component. The detailed immunohistochemical features are listed in Table 1.

**Table 1:** Immunohistochemical expression in the present case

Antibody	Classical component	High-grade component
ER/PR/HER2	(−)	(−)
SMA/ CK5/6/ p63/calponin	(−)	(−)
Collagen type IV	(−)	(−)
GCDFP-15	(−)	(−)
DOG1	(−)	(−)
INSM1/CgA/Syn/CD56	(−)	(−)
AAT/LYS/PASD	(+)	(−)
CK	(+)	(−)
EMA	(+)	(−)
E-cadherin	(+)	(−)
S100	(+)	(−)
SOX10	(+)	(−)
GATA3	(+)	(+), scattered
p53	(+), focally weak	(+), 80%
Ki-67	(+), 60%	(+), 95%
cyclinD1	(+), focally	(+), diffusely
Vimentin	(−)	(+), diffusely
LCA	(−)	(−)
MLH1/PMS2/MSH2/MSH6	(+)	(+)

MMR proteins: MLH1, PMS2, MSH2, and MSH6

### Molecular findings

Next‑generation sequencing (NGS) targeting all the exons of 769 cancer-related genes was performed separately on the classical and high-grade components of this case. The results (Table 2) revealed 23 gene mutations or copy number variants in the two components, of which 10 (43.5%) variants were identical in both components, including the mutations of *TP53*, *LMO1*, *MDC1*, *MSH3*, *KMT2D*, and *CCND3* and the copy number gains of *CCND1*, *FGFR2*, *MYC*, and *IDH1*. Remarkably, each mutant allele frequency (MAF) or the copy number (CN) of these shared variants was higher in the high-grade lesion. Moreover, *KMT2C* (c.161 + 1G > A) was identified in the classical component, whereas *KMT2C* (c.250 + 1G > A) was identified in the high-grade component. Both the classical and high-grade components were microsatellite-stable with tumor mutation burden of 8.78 and 5.85 mutations/Mb, respectively. No gene fusions or rearrangements were detected in both components.

**Table 2:** Identical and different gene mutations or copy number variants detected in different components of the tumor

	Gene	Variant type	MAF (%)/CN (n)	
			Classical lesion	High-grade lesion
**Identical gene mutations or copy number variants detected in both components**				
1	*TP53*	c.884_909del26, p.P295Hfs*2 frameshift	9.6	35.08↑
2	*LMO1*	c.32 C > T, p.P11L, missense	58.3	95.67↑
3	*MDC1*	c.3871 C > G, p.L1291V, missense	51.15	54.56↑
4	*MSH3*	c.1778G > A, p.R593Q, missense	36.5	37.65↑
5	*KMT2D*	c.13,003 C > T, p.Q4335*, nonsense	27.33	90.59↑
6	*CCND3*	c.29G > A, p.S10N, missense	45.16	62.85↑
7	*CCND1*	11q13.3, CN gain	3.24	4.45↑
8	*FGFR2*	10q26.13, CN gain	3.6	5.79↑
9	*MYC*	8q24.21, CN gain	4.61	6.49↑
10	*IDH1*	2q34, CN gain	3.36	3.52↑
**Different gene mutations or copy number variants detected in both components**				
1	*KMT2C*	c.161 + 1G > A, -	1.14	-
2	*ALOX12B*	c.392 A > T, p.E131V, missense	6.22	-
3	*KDM5A*	c.2225G > Ap.W742*, nonsense	5.07	-
4	*PIK3CD*	c.2608 C > Tp.R870*, nonsense	2.27	-
5	*POLE*	c.2974G > Ap.A992T, missense	2.18	-
6	*KMT2C*	c.250 + 1G > A, -	-	1.03
7	*PAK5*	c.686 A > T, p.D229V, missense	-	3.67
8	*CDH1*	16q22.1, CN loss	-	1.04
9	*CDK6*	7q21.2, CN gain	-	4.63
10	*CHEK1*	11q24.2, CN loss	-	1.21
11	*HGF*	7q21.11, CN gain	-	3.14
12	*MLH1*	3p22.2, CN loss	-	1.15
13	*FOXP1*	3p13, CN gain	-	3.09

Furthermore, both components had their unique genetic variants. *ALOX12B*, *KDM5A*, *PIK3CD*, and *POLE* mutations were identified in the classical component. *PAK5* mutation; CN loss of *CDH1*, *CHEK1*, and *MLH1*; and CN gains of *CDK6*, *HGF*, and *FOXP1* were identified in the high-grade component. All identical and different gene mutations or copy number variants detected in different components of the tumor are listed in Table 2.

The patient was finally diagnosed with breast AciCC with a high-grade solid component. After surgery, she received eight cycles of adjuvant chemotherapy (epirubicin combined with cyclophosphamide) and radiotherapy. She was followed up closely with periodic rechecks. Currently, she is alive for 43 months with no metastases or recurrences.

## Discussion

Although the morphological and molecular characteristics of several salivary gland-type breast tumors overlap with those of their salivary gland counterparts, including secretory carcinoma [11] and adenoid cystic carcinoma [12], breast AciCC was identified as genetically different from salivary AciCC. Sanger sequencing [3] of 10 breast and 20 salivary AciCC cases revealed that breast AciCC harbored *TP53* (8/10, 80%) and *PIK3CA* (1/10, 10%) mutations, whereas salivary AciCC harbored none of these mutations (0/20, 0%). Moreover, recurrent genomic rearrangement (4;9) (q13;q31) was identified in salivary AciCC, which results in the upregulation of the nuclear transcription factor NR4A3 that can be detected using the immunohistochemical marker NR4A3. Nevertheless, such gene rearrangement was not identified, and NR4A3 staining was consistently negative in breast AciCC [4, 13]. The molecular alterations identified in breast AciCC include *TP53*, *PIK3CA*, *MTOR*, *CTNNB1*, *BRCA1*, *ERBB4*, *ERBB3*, and *INPP4B* mutations; *TC2N-FBLN5* intrachromosomal fusion gene; and focal amplification of 12q14.3–12q21.1 of *MDM2*, *HMGA2*, *WIF1*, *FRS2*, and *PTPRB* [14, 15]. Among these, *TP53* was the most commonly mutated gene in breast AciCC [8, 9]. *TP53* mutation was also detected in the present case.

Genetic studies have shown that MGA and atypical MGA may be a part of the same spectrum of lesions harboring frequent *TP53* somatic mutations and MGA or atypical MGA with associated carcinoma might being the nonobligate precursor lesion of breast AciCC [8]. In the present case, no MGA or atypical MGA components were observed though comprehensive immunohistochemical and histological evaluation. This is perhaps because there existed other precursor lesions for breast AciCC besides MGA or atypical MGA. It is necessary to accumulate additional cases for further investigation. Moreover, carcinomas developing in MGA often have a metaplastic carcinoma component [16], whereas no metaplastic carcinoma components were identified in our case despite detailed pathological sampling and observation.

In the present case, the transitional histology of the classical breast AciCC component and high-grade solid component was observed in the same mass of left breast, and based on the distribution of both components, they were presumed to be the same tumor with different stages of differentiation. Nevertheless, the morphological and immunohistochemical features varied significantly between the two components. This leads us to the concept “high-grade transformation” or “dedifferentiation,” which has been previously used in parotid AciCC that is characterized by the abrupt transformation or progression of low-grade carcinoma into high-grade carcinoma [17, 18]. In salivary AciCC with high-grade transformation, the high-grade component was composed of solid cribriform patterns of neoplastic cells with large vesicular pleomorphic nuclei, prominent nucleoli, frequent mitoses, and a higher Ki-67 labeling index [19]. Moreover, the high-grade component of salivary AciCC was characterized by strong nuclear staining for cyclin D1, whereas classical diagnostic immunophenotypic markers, such as S100, AAT, and LYS, were absent [18], similar to that in the present case.

Furthermore, CN loss of *CDH1* and CN gain of *CDK6* were detected in the high-grade lesion. The *CDH1* gene located on 16q22.1 encodes a cell adhesion protein, E-cadherin, that plays a vital role in gland formation, cell differentiation, polarity, and maintaining the integrity of epithelial cells [20]. Studies have shown that the abnormality of *CDH1* gene and the loss of expression of membrane E-cadherin are common in the lobular carcinoma of the breast [21, 22]. However, recent studies showed that 21% of invasive ductal carcinomas also exhibited the CN loss of *CDH1* and 27% of high-grade invasive ductal carcinomas exhibited reduced or loss of E-cadherin membranous expression [23]. The loss or reduced expression of E-cadherin may result in cellular dedifferentiation and facilitate cancer invasion and metastasis in breast cancer [24]. Furthermore, cyclin-dependent kinases 4 and 6 (CDK4/6) play a significant role in regulating cell-cycle progression from the gap phase to the DNA synthesis phase [25]. Therefore, in the high-grade lesion of the present case, the CN loss of *CDH1* and the CN gain of *CDK6* may partially explain the poor morphological differentiation of the high-grade lesion or the dedifferentiation from classical to high-grade components.

## Conclusions

Overall, we have described in detail the histopathological and genetic features of a breast AciCC with classical low-grade and high-grade components. The growth pattern, loss of immunophenotypic markers, and complex genetic variants were identified in the high-grade lesion, which may provide a morphological and molecular basis for further investigating the possible molecular mechanisms underlying high-grade lesions in breast AciCC.

## Data Availability

No datasets were generated or analysed during the current study.

## Abbreviations

AciCC: Acinic cell carcinoma
TNBC: Triple-negative breast cancer
AAT: α1-antitrypsin
LYS: Lysozyme
EMA: Epithelial membrane antigen
S100: S100 protein
CK: Cytokeratin
cell cycle protein D1: Cyclin D1
NGS: Next‑generation sequencing
CN: Copy number
MGA: Microglandular adenosis
ER: Oestrogen receptor
PR: Progesterone receptor
HER2: Human epidermal growth factor receptor 2
SMA: Smooth muscle actin
CK5/6: Cytokeratin 5/6
GCDFP-15: Gross cystic disease fluid protein 15
DOG1: Delay Of Germination 1
INSM1: Insulinoma-associated protein 1
CgA: Chromogranin A
Syn: Synaptophysin
LCA: Leukocyte common antigen
MMR: Mismatch repair
PASD: Periodic acid–Schiff-diastase
GATA3: GATA-binding protein 3
MAF: Mutant allele frequency
CN: Copy number
CDK4/6: Cyclin-dependent kinases 4 and 6
